# On the Microscopic Texture of Cancer

**Published:** 1845-09

**Authors:** 


*On the Microscopic Texture of Cancer.*—M. Desormeaux has recently published a valuable inaugural dissertation, entitled *Recherches sur la theorie elementaire de la production des tissus occidentels*, in which he has given an excellent summary of all the recent researches on the intimate structure of cancerous formations.

Müller, and (since the publication of his writings) most other pathologists, have arranged these morbid growths into two great families or groups, viz. the Encephaloid and Scirrhous. Of the former he makes the following three subdivisions.

1. Carcinoma medullare, *in which there is a predominance, in the medullary mass, of round globules over loose fibrous tissue.* The globules are of various sizes; but the smallest are larger than pus-corpuscles. Each contains a granular substance or nucleus within. They are very similar, in many respects, to those of common Cancer, and of reticulated Carcinoma or Scirrhus.

2. Carcinoma medullare, *consisting of pale, elliptic, non-elongated Corpuscles, and of a fundamental cerebriform mass.* These corpuscles are usually twice or three times as large as the globules of the blood. There is never any appearance of fibres proceeding from their surface, and they rarely exhibit any traces of nuclei within them.

3. Carcinoma medullare *with fibrated or fusiform corpuscles.* This species of Encephaloid structure has at times, on laceration, a sort of fibrous aspect, when the fusiform corpuscles are arranged in a somewhat determinate direction. According to the direction which they assume, the morbid mass will present a radiated or a tufted appearance. In many cases, indeed, their directions are so various that the lacerated surface exhibits no trace of fibres any where. The fusiform corpuscles are sometimes nucleated; at other times they contain granular points, but without distinct nuclei. They are elongated on one or two sides into fibres of different lengths. They may be considered as cells that are arrested at the period of the process of transition from the cellular to the fibrous condition.

The three forms of the disease now described may (most probably) be regarded as so many degrees or stages in the development of the same tissue; these successive stages being characterised, 1, by rounded nucleated globules; 2, by elongated oviform globules, which are either non-nucleated, or indistinctly so; and, 3, by fusiform globules.

These several kinds of globules may be regarded as so many successive epochs of evolution, through which a *cell* must pass before it can become a fibre. Thus we find that, in an Encephaloid mass, there is the same transformation of the primitive elements as occurs in many normal tissues—with this difference only, that the process of evolution is not complete, being arrested before the fibrine is perfectly formed. There is a perfect analogy in their mode of formation. The essential element of an encephaloid tumour is the presence of cells. In some cases the entire mass is composed of them, placed one alongside of the other, but without having any perceptible bond of union; while in others, there is a network of fibrous or cellular tissue interposed between the cells. When this fibrous tissue prevails, the Encephaloid then approaches in character to the Scirrhous structure. In the latter, the existence of the two elements—cells and fibres—is always more distinctly marked than in the former. The fibres are often quite perceptible by the naked eye. Sometimes they are lengthened and run parallel to each other; at other times, they form rounded capsules, within which the globules are contained. As in the case of the newly-formed fibres of the cellular tissue, so those of a scirrhous formation are destroyed by acetic acid, leaving nuclei or nucleated fibres behind. The fibres sometimes exhibit at different points a sort of varicose enlargement, within each of which a nucleus is found. This appearance is often observed in fibrous tumours (not genuine scirrhus) of the uterus and other parts.

In the *reticular Carcinoma* of Müller, the white network, which encloses the scirrhous globules in its meshes, is formed of round opaque granulations, three or four times as large as the blood-globules: they are occasionally agglomerated into round masses. The genuine Scirrhous tissue, of a pale greyish colour, is composed of globules that, on the whole, resemble those of the first stage of Encephaloid formation. These globules are either round or somewhat oval: along with them we find free *nuclei* with their *nucleoli*.—(Vogel.)

From a variety of observations we may reasonably conclude that the cells of Scirrhus are formed around the nuclei of which M. Vogel speaks; their contents are at first granular and almost opaque. When the process of softening commences, the granulations disappear, the globules become transparent, and within them are formed new cells, which at first are few in number, aud gradually multiply, until they entirely fill the parent cell. M. Valentin, who, in part at

52*

least, admits this account of the progress of the cells, says, that the parent cells eventually burst and discharge their cellules: in this way we may account for the presence of young free cells in scirrhous formations that have become softened.

The intercellular substance seems to undergo certain modifications corresponding with the evolution of the cells; the granulations or granular points, which it often contains, usually disappear, and it becomes limpid, while at the same time the space, which it occupies, is diminished by the enlargement and multiplication of the cells.

The fibrous network does not appear to follow in its alterations the development of the cells: it may remain firm and resisting, while the cells are far advanced in their evolution. Even when a scirrhous tumour has become completely softened, this tissue sometimes forms shreds that retain their original character.

In *alveolar Cancer*, the basis of the morbid tissue consists of white fibres and lamellæ, which cross and intercross with each other, intercepting between the meshes thereby formed limpid cells, either closed or communicating with each other, of various sizes from that of a grain of sand to that of a large pea, and filled with a transparent gelatinous substance. In this substance there are cells, and these cells contain other cells more minute. The smallest of these cells exhibit at one point of their parietes a distinct dark-yellowish nucleus, and sometimes also many free and unattached granules floating within them. To this species M. Müller refers the *gelatiniform* and *areolar cancers* of Laennec and Cruveilhier. The cells of this species of the disease appear to be only an advanced or more mature degree of the cells of Scirrhus.—*Lond. and Ed. Month. Journ., from Journal de Chirurgie de M. Malgaigne.*

